# On the “Life-Likeness” of Synthetic Cells

**DOI:** 10.3389/fbioe.2020.00953

**Published:** 2020-08-26

**Authors:** Luisa Damiano, Pasquale Stano

**Affiliations:** ^1^Research Group on the Epistemology of the Sciences of the Artificial (RG-ESA), Department of Ancient and Modern Civilizations, University of Messina, Messina, Italy; ^2^Laboratory of Bio-Organic Chemistry, Department of Biological and Environmental Sciences and Technologies (DiSTeBA), University of Salento, Lecce, Italy

**Keywords:** synthetic biology, synthetic cells, artificial cells, autopoiesis, self-organization, Turing test, molecular communications, life-likeness

One of the most exciting and rapidly expanding research area in contemporary science is the bottom-up construction of artificial cell-like systems, also known as synthetic cells. Such approaches are part of the synthetic biology research paradigm, which equates to understanding means constructing. Accordingly, these artificial systems are considered able to generate new knowledge based on explorative procedures that are complementary to traditional scientific investigations. Constructing synthetic cells aims at understanding the emergence of life from scratch at the cellular level, modeling chemically primitive cells for unveiling origins-of-life mysteries, and developing radically new biotechnological tools for medical, industrial, and research applications. The following article is dedicated to one of the most compelling open questions emerging from the rapid improvement of synthetic cell technology: “life-likeness” of synthetic cells. Based on prior work, we promote an ‘organizational approach’ to the assessment of life-likeness, and, coherently, we propose the transition from behavioral assays, like the Turing test, to systemic strategies, based on concepts such as organization, complexity, networks, and emergence.

## 1. Understanding by Constructing: Cell Models in the Age of Synthetic Biology

The current age of synthetic biology (SB), for the first time, makes possible novel ways of understanding the emergence of cellular life by means of “constructive” or “synthetic” approaches, also known as “bottom-up” or “understanding-by-building” methods. The operative paradigm is based on the assembly of molecular components into a spatially organized structure that resembles and behaves like a living cell, even if at a minimal complexity level. *Synthetic cell technology* is an innovative blend of microcompartment technology (in particular, but not exclusively, liposomes), cell-free systems, microfluidics, and numerical modeling. The ever increasing number of experimental reports referring to “synthetic cells” (SCs), “artificial cells,” and “protocells” shows that the goal of constructing cell-like systems in the laboratory is now within the experimental reach. Such advances trigger new stimulating questions about practical and conceptual issues referring to SC construction, such as organizational issues (complexity, life-likeness), materials (primitive-like, modern biochemicals, fully synthetic), goals (basic or applied science). Motivated by the current discussions about the “science of the artificial” (Cordeschi, 2002; Damiano et al., 2011), here we aim to shortly comment on the first issue, in particular about life-likeness, with the goal of stimulating an open discussion in the community.

## 2. How to Quantify SC Life-Likeness?

### 2.1. The Imitation Game

**Figure 1:**
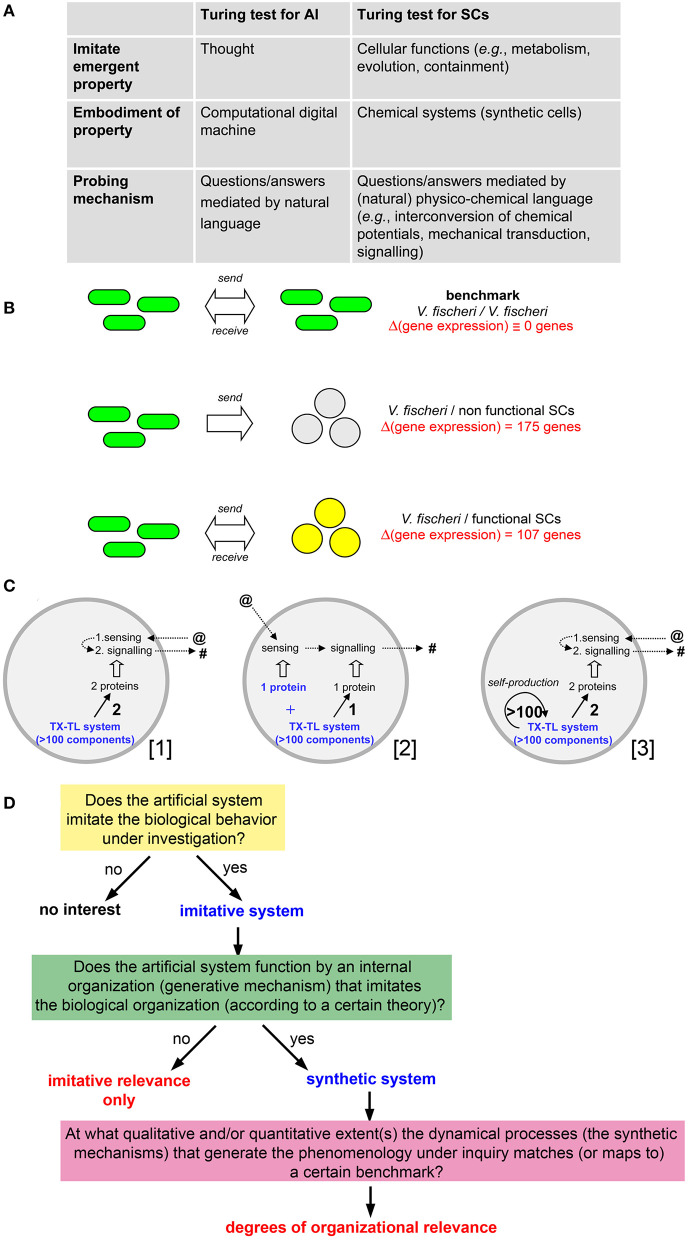
**(A)** Comparison of Turing tests for intelligence and life, taken from Cronin et al. (2006). **(B)** Graphical summary of the method used by Lentini et al. (2017) to quantify life-likeness of SCs capable of bidirectional chemical communication with *V. fischeri*. (Top) The biological communication between when *V. fischeri* cells is taken as benchmark against which the synthetic/natural communication is contrasted. (Middle) When *V. fischeri* cells send a signal to SCs that are not able to reply, a change in the expression levels of 175 *V. fischeri* genes is observed. (Bottom) When *V. fischeri* cells send a signal to SCs that are able to reply with a response signal, a change in the expression levels of 107 *V. fischeri* genes is observed. Therefore, SCs behavior is capable of offsetting 68 (= 175 − 107) differences in *V. fischeri* gene expression levels, recovering the 39% (= 68/175) of the biological behavior. **(C)** Three examples of SCs capable of establishing the kind of molecular communication with *V. fischeri* of the same type as described by Lentini et al. (2017). (Left) The actual experimental system made in the laboratory: TX-TL machinery produces two proteins capable of sensing the primary signal (sent by the bacteria), and, as response, produces a secondary signal (sent to the bacteria); this SC is based on the expression of two genes. (Center) A hypothetical SC—simpler than the one shown on the left, where the receptor is not produced by the TX-TL machinery, but it is added from the beginning as ready-to-work protein; this SC is based on the expression of one gene. (Right) A hypothetical SC—very complex and currently out-of-reach—where TX-TL components (>100) are all produced by the TX-TL machinery itself, together with the two proteins needed for communication (this SCs is based on the expression of >100 genes). The three SCs clearly have different complexity ([3] >> [1] > [2]) and different life-likeness, but they would result in the same 39% value when evaluated by a behavioral assay (as in **B**). **(D)** A possible workflow diagram—inspired from Damiano et al. (2011)—for the evaluation of SC life-likeness based on the measure of “organizational relevance.” The organizational relevance is evaluated after a first screening for the “imitative relevance.” The question is how to devise a method to map the dynamical processes occurring within the artificial system (i.e., the synthetic mechanisms that generate the behavior under investigation) with a benchmark organization, stemming from a reference theory. This study suggests that systemic theories of life, such as autopoiesis, (M,R)-systems and relational biology, or chemoton are best candidate for it, but it left to future investigation the screening and the selection of the best criteria for their application.

In 2006 the members of the project CHELLnet (2005–2009) published a thought-provoking position paper focused on the relevant yet elusive question of *how much alive a SC is*. The Authors start the discussion by remarking that no universally acceptable definition of life is available, while the progresses in SB are going to allow the construction of cell-like systems of increasing complexity. How to determine, then, their life-likeness? Following Turing's proposal for artificial intelligence (AI), the answer was a sort of extension of the Turing test [or “imitation game” (Turing, 1950)] to the realm of SCs, in the framework of the analogies indicated in Figure 1A.

In the case of AI, the long-debated question “can machines think?” was re-defined by Turing based on an operational scenario consisting in a machine that *imitates* the act of thinking, in such a way that a human interrogator, using a natural language, cannot distinguish between a machine's answer and a person's answer. In the SB context, the question becomes “are SCs alive?” and thus it can be re-defined by an operational scenario where SCs do perform well (or do not) in a sort of cellular imitation game. The provided example refers to the SCs capacity of establishing a chemical communication with natural biological cells—in the spirit of the original Turing test.

The 2006 paper on the SC Turing test (and the CRAACC workshop held in the same year in Venice, hosted by the ECLT) were illuminating and anticipated new directions in the SC research that we are starting to approach now. Although a real Turing test for SC may still seem unrealistic, these studies have the merit of having raised very intriguing questions.

### 2.2. Experimental Results

The original idea of applying the Turing test to SCs generated a first pioneer report a few years later. Ben Davis and collaborators published an experimental paper on chemical communications between SCs and biological cells in 2009 (Gardner et al., 2009). The so-called *formose* reaction was carried out inside liposomes. Some of the products, after escaping from the liposome and reacting with borate in the medium, reached *Vibrio harveyi* bacteria and activated a response.

SC technology, however, allows the construction—sometimes microfluidics-aided (Weiss et al., 2018)—of more sophisticated and programmable SCs based on the control of gene expression. These SCs can be seen as general-purpose systems that can be interfaced with biological cells, exchange signals, and be engaged in communicative interactions. An early elaboration of this scenario was obtained by merging pre-existing ideas from SB, bio-engineered nanofactories, and molecular communication networks (Luisi et al., 2006; Leduc et al., 2007; Nakano et al., 2011, 2013; Stano et al., 2012). Experimental results on SCs communicating with other SCs or with biological cells appeared more recently (Lentini et al., 2014, 2017; Adamala et al., 2017; Ding et al., 2018; Niederholtmeyer et al., 2018; Rampioni et al., 2018; Tang et al., 2018; Dupin and Simmel, 2019; Joesaar et al., 2019) showing that establishing chemical communication is now within the reach of several laboratories. Details on the molecular circuitry employed in these studies have been reviewed by Rampioni et al. (2019).

One of these studies is quite relevant and deserves a close attention, because it reports for the first time a *bidirectional* SCs-biological cells communication (Lentini et al., 2017), thus realizing, in a minimal form, the concepts envisioned in the above-mentioned 2006 paper (Cronin et al., 2006). In particular, SCs based on gene expression were able to perceive and decode a primary chemical signal sent by *V. fischeri*, then reply by synthesizing a response signal sent back to, and perceived by, *V. fischeri*. Results were explicitly presented from the viewpoint of the cellular Turing test and attracted the attention of science press (Urquhart, 2017).

Based on the two-way communication between SCs and bacteria, the life-likeness of the SCs employed in that study was estimated, consistently with the seminal idea of the Turing test mentioned above. It is informative, then, commenting on the proposed approach, in light of the present discussion. The strategy is based on measuring the *bacterial* gene expression profile when a successful bidirectional communication channel is established with SCs (see Figure 1B), estimating the SC life-likeness as 39% (technical details in Figure 1B). The result is at the same time exciting and debatable. The bidirectional communication pattern described above is generated by SCs that just express two genes: a receptor for sensing the signal molecules sent by the bacterium, and a synthase for producing the response signal molecules. How can a system of such (low) complexity displaying 39% life-likeness?

Mansy et al. carefully discussed their conclusions, with the correct intuition that, in addition to the two expressed genes, one should also consider the about 100 macromolecular elements of the transcription-translation (TX-TL) machinery that operate in the background, all essential for expressing the two genes. Accordingly, the value of 39% actually was considered as a proxy for the ratio between the number of genes of the TX-TL machinery and the about 200 genes recognized as the so-called *minimal genome* (Mushegian and Koonin, 1996; Gil et al., 2004; Islas et al., 2004; Forster and Church, 2006), essential for a minimal cellular life. In other words, it is suggested that the 39% value actually refers to a *hypothetical* SCs that could produce all TX-TL macromolecules and, consequently, the two proteins which are functional for the experimentally observed bidirectional molecular communication.

It is important to remark that the hypothetical SCs described above are not yet experimentally accessible, and they would be certainly much more complex (and more life-like) than the actual SCs used in that study. However, and this is the key point of the discussion here—even if that sort of SCs would exist, or other similar system of lower complexity, any analysis based on the gene expression of the *receiving V. fischeri* cells would still have 39% life-likeness.

This conclusion results from monitoring the differences induced in the biological partner for inferring how the synthetic partner *behaves*, irrespective of the structure and the functioning of the synthetic partner (by “functioning” here we mean the mechanisms that generate its behavior, i.e., its organization). Indeed, the gene expression profile of the receiving biological cells would not change if three different SCs with different complexity would engage the same communication with biological cells, as shown in detail in Figure 1C.

### 2.3. Limitations of the Imitation Game

The Turing test for AI focuses only on the “recognizability,” by a biological system, of some phenomenology (behaviors) produced by an artificial system, without referring to the biological plausibility of the underlying mechanisms operating within the artificial system. Shannon and McCarthy (1956), and later Block (1981), among others, presented arguments against the purely behavioral approach expressed by the Turing test. In particular it has been claimed that a further *non-behavioral* criterion, referring to the manner in which the artificial system works, is needed for a proper qualification of the artificial agent. In the mentioned SCs/*V. fischeri* communication example, the life-likeness of the artificial system is inferred indirectly, by measuring the response achieved in the biological system. The manner in which the artificial system works is not considered, leading to the inconsistencies mentioned above (Figure 1C).

The definition of SC complexity and life-likeness therefore require further elaborations, and the previous considerations suggest to focus on non-behavioral criteria. The original intention of the Turing test was to assess AI without referring to any theory about intelligence functioning. However, assessing life at the minimal unicellular level can be soundly based on several descriptions, disclosed chemical mechanisms, and systemic/synthetic theories. The latter, in particular, can provide a useful starting block for discussing SC life-likeness beyond behavioral imitation.

### 2.4. The Organizational Perspective

As soon as SC behavior is deliberately skipped, SC life-likeness can be advantageously assessed by their *organization*, despite the fact that SCs are material entities existing in the chemical domain and discussing their *structure* is obviously tempting^1^. SC organization can be conveniently discussed within the Rosen relational theory of (Metabolism,Repair)-systems (Rosen, 1958, 1991; Letelier et al., 2003), the Maturana-Varela autopoiesis (Varela et al., 1974; Maturana and Varela, 1980; Luisi, 2003), and the Gánti's chemoton (Gánti, 2003). These systemic theories focus on the organization, depicting the relations between the elements and the transformations of components within a system, and describe life as a property emerging not from specific elements, but from a peculiar type of organization (Bich and Damiano, 2012).

How to move, then, from mere behavior, on which the Turing test is focused, to the patterns of generative mechanisms? With this regard, the *sciences of the artificial* (Simon, 1996; Cordeschi, 2002) can help significantly. These include important fields like cybernetics, robotics, artificial intelligence, artificial life, and most recently, synthetic biology, all having in common the construction of artifacts based on the so-called synthetic (or constructive) approach. For example, within the context of the epistemology of the sciences of the artificial, the relevance of artificial models of biological and cognitive processes has been recently discussed in an innovative manner (Damiano et al., 2011), based on an organizational approach to the scientific characterization of life that re-proposes the “life = cognition” thesis (Bich and Damiano, 2012)^2^. In Damiano et al. (2011) the authors argues that hardware, software and wetware artifacts can have different types of relevance, defined by the combination of two criteria, respectively assessing (a) an artificial system's capability of recreating the phenomenology typical of the natural process under study, and (b) the system's capability of reproducing, in its organization, the organizational mechanisms that in nature generate that phenomenology. In this perspective, an artifact designed for passing the AI Turing test by merely imitating a certain cognitive behavior is recognized to have a purely imitative relevance, and, in this sense, is considered less relevant, as a synthetic model of the target cognitive behavior, than an artifact based on plausible organizational mechanisms generating that behavior.

When translated in the current discussion, the above-mentioned insightful argument leads to an interesting direction. In particular, a distinction can be made between mere “imitative” relevance (low life-likeness) and deeper “organizational” relevance (high life-likeness) according to the scheme shown in Figure 1D. The criterion of organizational relevance (stratified after the imitative one) can measure the SC life-likeness if a suitable “benchmark,” directly stemming from a theory of reference, is available.

Autopoiesis has inspired SCs research from its beginning (Luisi and Varela, 1989; Walde et al., 1994; Luisi, 2003) and thus it appears a suitable theory of reference for the conceptual and operational definition of organizational criteria in SC research. Let us recall that the autopoietic organization is defined as:

(…) a network of processes of production (transformation and destruction) of components that produces the components which (i) through their interactions and transformations continuously regenerate and realize the network of processes (relations) that produced them; and (ii) constitute (…) a concrete unity in the space in which they (the components) exist by specifying the topological domain of its realization as such a network (Maturana and Varela, 1980, p. 79).

A possible quantification of SC life-likeness can be based on the comparison between the actual SC organization and the minimal autopoietic organization. The latter, however, is not specified, there is nothing as a “standard autopoietic network” to refer to. This depends, ultimately, by the actual realization of the autopoietic system, and thus by the type of elements contributing to the autopoietic network, as well as by the foodstuff supplied by the environment^3^. Autopoietic systems can have in principle different realizations, provided that their autopoiesis and their self-bounding as unity is maintained. For example, if SCs are realized—as it often happens—with familiar biochemical molecules (DNA, RNA, proteins, lipids, …), their minimal complexity is determined (and constrained) by the known biochemistry, because the autopoietic network needs to generate all SC components.

Let us imagine, then, being able to devise a proper and suitable benchmark autopoietic network, which can be realized, at least in principle, in the chemical domain, and endowed with the above-mentioned requirements. The next step would be the selection of criteria for mapping the actual SC organization with the autopoietic benchmark (such a question does not need to be answered here). Life-likeness would result from a comparison between the two organizations (SC vs. autopoietic). Consider for example the above-mentioned synthetic/natural bidirectional communication (Lentini et al., 2017). The benchmark autopoietic network would correspond to a hypothetic SC that produces all components of its organization (e.g., about 100 macromolecules), whereas it can rely on the full set of low MW compounds freely available in the environment. Such an organization consists of a self-bounding constraint and of hundreds of confined transformations, with the possibility of importing and exporting small molecules. Vice versa, the actual SC has been able to produce only a small number of the network components. Comparing these two networks leads estimate SC life-likeness as a very small number (<1%?), which seems more reasonable than the 39% estimated on the basis of the behavioral Turing test.

## 3. Outlook and Open Questions

It could be argued that referring to a theory (e.g., autopoiesis, or other systemic theories) to measure the SC life-likeness actually brings back the problem of definition of life into the question of determining the life-likeness: exactly against the general motivation behind the Turing test. While such a criticism should be seriously considered, there are two counter-arguments that can be taken into account. The first is that biological life, especially at the level of single cells, is more understood that human intelligence. Therefore, it is not unrealistic to approach life-likeness as discussed above. Secondly, referring to the systemic perspective is very attractive because theories, such as autopoiesis, (*M,R*)-systems, and chemoton do not attempt defining life by a list of attributes, but they provide high-level relational descriptions about how living systems function. Accordingly, this move will lead to fecund landscapes where the entire technical and conceptual toolbox of complex adaptive systems theory (complexity theory) can be applied to next steps of SC research, further enriching this rapidly evolving field.

## Author Contributions

PS conceived the research and wrote the manuscript. LD contributed to the epistemological aspects. All authors contributed to the article and approved the submitted version.

## Conflict of Interest

The authors declare that the research was conducted in the absence of any commercial or financial relationships that could be construed as a potential conflict of interest.

## Footnotes

^1^Here we refer to the molecular structure of the elements constituting the SCs. In principle, different chemicals can lead to networks of transformations that map into the known biological ones, when causal relationships are considered [an example is the “Rasmussen protocell” (Rasmussen et al., 2004)]. Structure is instead relevant in topological sense, i.e., as a determinant of the unity of SCs in space and time, and must emerge from the physical self-organization of the system's components. For example, non-self-bounded systems should be excluded.

^2^We refer to the autopoietic description of the biological domain introduced by Maturana and Varela (1980), whose innovative definition of life will be discussed later in this paragraph. Autopoiesis rejects the classical analytical approach organizing scientific definitions of life as lists of properties considered essential for living systems. Recognizing that any such list may always be susceptible to criticism and revision, and be rejected by part of the scientific community, autopoiesis proposes a synthetic approach to the definition of life. This consists in directing the definitional effort not toward the identification of the essential properties of living beings, but toward the determination of an organizational mechanism capable of generating, from a plurality of components, the minimal living cell and, on this basis, all living phenomenology as we know it (Bich and Damiano, 2007). This mechanism, which Maturana and Varela characterized as the “autopoietic organization,” is considered by them to be capable to create all biological processes and features, including the properties typically listed in analytical definitions of life – autonomy, production of the molecular components (including the replication of information-carrying molecules), growth-and-division (reproduction), sensing the environment, etc. In this sense, from the autopoietic point of view, all the properties we can scientifically ascribe to living systems are grounded in this specific organizational mechanism (the system's autopoiesis), which generates living processes as cognitive processes, and is responsible for the emergence of intelligence in higher-level biological domains (Bich and Damiano, 2012).

^3^In a chemically “rich” environment, for example, autopoietic systems can be simpler than the one established in a chemically poor one (endosymbionts and parasites, living inside other cells, illustrates well this principle).
